# Research on the Additional Secondary Phase Factor for Automatic Identification System Signals Transmitted over a Rough Sea Surface

**DOI:** 10.3390/s18020617

**Published:** 2018-02-17

**Authors:** Xiaoye Wang, Shufang Zhang, Xiaowen Sun

**Affiliations:** Information Science and Technology College, Dalian Maritime University, Dalian 116026, China; sfzhang@dlmu.edu.cn (S.Z.); sunxiaowen_1984@163.com (X.S.)

**Keywords:** automatic identification system (AIS) signals, rough sea surface, additional secondary phase factor (ASF), reflection propagation, diffraction propagation

## Abstract

This paper investigates the Additional Secondary Phase Factor (ASF) characteristics of Automatic Identification System (AIS) signals spreading over a rough sea surface. According to the change of the ASFs for AIS signals in different signal form, the influences of the different propagation conditions on the ASFs are analyzed. The expression, numerical calculation, and simulation analysis of the ASFs of AIS signal are performed in the rough sea surface. The results contribute to the high-accuracy propagation delay measurement of AIS signals spreading over the rough sea surface as, well as providing a reference for reliable communication link design in marine engineering for Very High Frequency (VHF) signals.

## 1. Introduction

With the rocketing development of the world maritime adventure and the rapid progress of shipbuilding technology, all countries in the world are paying more and more attention to life at sea and navigation safety [1]. The International Maritime Organization (IMO) has set out a list of technologies that must be met by an electronic positioning system during the various voyage phases [2], which sets higher technical requirements for shipborne navigation equipment. As one of major land-based radio navigation systems, the Automatic Identification System (AIS)—in the very high frequency—is a communication system for maritime information transition, such as Maritime Mobile Service Identity (MMSI), position, course, and speed [3]. Shipborne AIS equipment is mandatorily installed according to the IMO and major coastal states, and regions in the world have established comparatively perfect AIS shore-based systems [4]. Presently, highly reliable and robust position, navigation and time (PNT) data are primarily provided by the Global Navigation Satellite System (GNSS) [5]. Due to some intentional or unintentional interferences, such as signal abnormity, signal failure, signal blocking, spectrum competition, and human-made interference [6], relying solely on GNSS has been identified as a potential safety hazard [7,8]. Once GNSS fails, the shipborne navigation equipment will not receive correct maritime navigation information, which presents a serious threat to navigational safety [9]. The IMO has explicitly stated that all ships should mandatorily install space-based and land-based backup systems [10,11,12]. AIS is an alternative future land-space navigation system [9], which could provide PNT information without depending on other navigational sensors [3]. Research on a land-based positioning system based on AIS base stations has been conducted since 2012 by the Navigation Institute of Dalian Maritime University in Dalian, China, where the authors are located. The aim of this research is to achieve high-precision positioning by using VHF signals to measure the transmission delay of an AIS signal. The AIS signal transmission on the sea surface is seriously influenced by the transmission medium. By considering the sea surface as one of the transmission mediums, the SF and the ASF are collectively referred to as the ASF in the context of this paper, and is the sum of the additional phase delay caused by the transmission medium that is different from the atmosphere. Without the additional secondary phase factor (ASF) correction, the positioning accuracy of the verification system for the AIS Autonomous Positioning System (AAPS) was more than 100 m (2σ) with a geometric dilution of precision (GDOP) of less than 1.5 [3]. Extensive research on the ASF correction is common in Loran-C and eLoran systems at home and abroad, whose frequency and transmission mode are different from the AIS system. Therefore, the existing results could not be completely copied. The authors have been working on the ASF study of AIS signals transmitted over the sea surface and have developed an ASF correction system based on [13] and [14] as the theoretical basis. The system will correct, in real-time, the original measurement of the positioning receiver so that the corrected positioning accuracy could meet 10 m (95%) [3]. However, the ASF of AIS signals over a rough sea surface has not been calculated and as analyzed accurately and in detail in [13,14], the PNT information provided by the GNSS is more likely to be distorted in extreme weather. Once the shipborne equipment cannot receive a maritime navigation signal properly, there will be a potential threat to navigation safety [15,16]. Therefore, it is urgent to study the ASF characteristics of AIS signals over a rough sea surface. In this paper, the ASF characteristics of AIS signals over a rough sea surface are studied to obtain the ASF correction parameters of AIS signals in extreme weather, which could improve the positioning accuracy of AIS signals comprehensively. This work extends and supplements the results reported in [17].

Since the Earth is a sphere, the reflection surface of the AIS signal is no longer a plane, and the transmitting and receiving antenna heights and the plane reflection coefficient need to be corrected [18]. In the range of line-of-sight propagation, it is difficult to rigorously solve the reflection coefficient of an arbitrarily rough surface. This is because the actual surface is almost impossible to describe with finite parameters, except for some specifically ideal surfaces. Therefore, the actual surface could be approximately solved after being idealized [19]. The reflection coefficient of a rough sea surface is the product of the reflection coefficient of a smooth sea surface, the diffusion factor decreasing the reflection coefficient is caused by the curvature of the Earth and the correction coefficient of the sea surface roughness [19]. The reflection coefficient of the smooth sea surface is the Fresnel reflection coefficient, which is affected by the propagation distance, the heights of the transmitting and receiving antennas, the wavelength of the AIS signal, and the relative permittivity and conductivity of the sea. The diffusion factor could be obtained by formulas. The Miller-Brown model is commonly used for calculating the correction coefficient of the sea surface roughness [20,21,22,23]. When the sea surface is rough, the rolling waves will block the AIS signal propagation, and the AIS signal will continue to spread around the waves in a diffractive way [24]. The diffraction of a radio wave along the Earth’s surface is influenced by irregular topography [25], and the knife-edge obstacle diffraction model discussed in the ITU-R P.526-12 Recommendation can be used [26]. However, the Recommendation discusses only provide mathematical models of the diffraction loss and these models not suitable for research on the ASF characteristics of the AIS diffracted signal in this paper. In order to study the scattering and diffraction of the radar wave in the rough sea surface of the island, the expression of the knife-edge diffraction field strength is provided in [27]. In this paper, we derive and calculate the ASFs of the AIS diffracted signal on the rough sea surface according to the received field strength diffracted by a knife-edge obstacle in [27]. It can be deduced that the ASFs of AIS signals in a rough sea surface is complex and changeable, so it is necessary to study it in depth, and the results can serve as a reference for the design of other maritime VHF communication links [14]. As the signal of the maritime VHF communication links is vertically polarized in the practical application, the ASFs of AIS signals for vertical polarization is only studied in this paper. This paper considers the ASFs of the AIS received signal transmitted on the rough sea surface in the line-of-sight transmission mode and is organized as follows: In the line-of-sight transmission mode, the phases of the reflection coefficient on the rough sea surface and the ASFs of the AIS received signal are calculated by changing with the propagation distance, the seawater temperature, the sea salinity, and the wind speed in Section 2. Section 3 calculates the ASFs of the AIS diffracted signal changing with the wave height and the clearance parameter. The numerical results of the ASFs and the range positioning error corrections for the AIS received signal spreading over the rough sea surface are summarized in Section 4. Finally, some conclusions are put forth in Section 5.

## 2. The ASF of the AIS Reflected Signal

In the range of line-of-sight propagation, the AIS received signal is mainly composed of the direct signal and the reflected signal. Xiaoye [13] mainly studied the ASFs of the AIS reflected signal transmitted on the smooth sea and did not study too much about the ASFs of the AIS signal transmitted on the rough sea surface. In order to improve the AIS positioning accuracy to cope with the distortion of the received signal in the extreme weather, it is necessary to study the ASF characteristics of the AIS signal transmitted on the rough sea deeply and in detail. Considering the existence of the Earth’s curvature, this section calculates the equivalent heights of the transmitting and receiving antennas at first, gives the spherical emission diffusion factor and the ASF expression of the AIS reflected signal under the rough sea condition, and the changing trend charts and the numerical results of the ASFs of the AIS reflected signal are provided in the different seawater temperature, salinity, propagation distance, and wind speed conditions.

### 2.1. The Equivalent Antenna Heights

**Figure 1:**
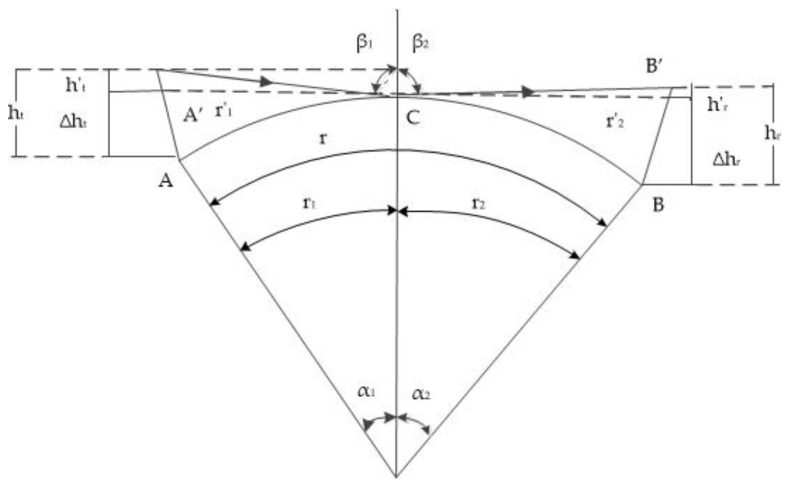
The ray geometry of the spherical sea surface.

Only when the wavelength is relatively long and the propagation distance is relatively close can the spherical ground be approximated as the ground plane [18]. Therefore, as the AIS signal belonging to the ultrashort wave, the influence of the spherical sea surface on the radio wave propagation must be considered. The ray geometry of the spherical sea surface [18] is shown in Figure 1, A, B are transmitting and receiving points, respectively. A’, B’ are equivalent transmitting and receiving points.$h_{t}$ and $h_{r}$ are the heights of the transmitting and receiving antennas. The equivalent heights of transmitting and receiving antennas are $h_{t}^{\prime }$ and $h_{r}^{\prime }$, respectively. C is the reflected point. *R* is the Earth’s radius. $\alpha_{1}$ and $\alpha_{2}$ are geocentric angles, and $\beta_{1}$ and $\beta_{2}$ are the incident angle and the reflected angle, respectively.

According to Figure 1:

(1) $$\begin{array}{l} h_{t}\approx h_{t}^{\prime }+\Delta h_{t}\\ h_{r}\approx h_{r}^{\prime }+\Delta h_{r} \end{array}$$

where:

(2) $$\begin{array}{l} r_{1}^{\prime }=\sqrt{{\left(R+\Delta h_{t}\right)}^{2}-R^{2}}=\sqrt{2R\Delta h_{t}+\Delta h_{t}^{2}}\\ r_{2}^{\prime }=\sqrt{{\left(R+\Delta h_{r}\right)}^{2}-R^{2}}=\sqrt{2R\Delta h_{r}+\Delta h_{r}^{2}} \end{array}$$

According to [18]:

(3) $$\begin{array}{l} \Delta h_{t}=\frac{r_{1}^{2}}{2R}\\ \Delta h_{r}=\frac{r_{2}^{2}}{2R} \end{array}$$

Therefore:

(4) $$\begin{array}{l} h_{t}^{\prime }=h_{t}-\frac{r_{1}^{2}}{2R}\\ h_{r}^{\prime }=h_{r}-\frac{r_{2}^{2}}{2R} \end{array}$$

In Equation (4), the units of $h_{t}^{\prime }$ and $h_{r}^{\prime }$ are m and the units of *r*_1_ and *r*_2_ are km.

### 2.2. The ASFs of the AIS Reflected Signal over the Rough Sea Surface

According to the conclusion from the [28], under the condition that the AIS signal sea surface transmitted on the sea by the line-of-sight mode, the field strength of the reflected signal at the receiving point is [28]:

(5) $$\begin{array}{cc} e_{r}\left(t\right) & =E_{r}\exp\left[j\left(\omega_{c}t-kr_{2}\right)\right]\\ & =\Gamma_{P}e_{0}\left(t\right)\exp\left[j\left(\omega_{c}t-k\Delta r\right)\right]\\ & =\Gamma_{P}E_{0}\exp\left[j\left(\omega_{c}t-kr_{1}-k\Delta r\right)\right]\\ & =\left|\Gamma_{P}\right|E_{0}\exp\left[j\left(\omega_{c}t-kr_{1}-k\Delta r-\varphi_{\Gamma_{P}}\right)\right] \end{array}$$

where $e_{r}\left(t\right)$ is the field strength of the reflected signal and $e_{0}\left(t\right)$ is the field strength of the direct signal. $\Gamma_{P}$ is the reflected coefficient for the rough sea surface. $\omega_{c}$ and k are the angular frequency and the wave number of the AIS signal, respectively. $r_{1}$ is the propagation distance of the direct signal. $r_{2}$ is the propagation distance of the reflect signal. Δr is the propagation difference between the direct path and the reflect path. $\varphi_{\Gamma_{P}}$ is the phase of the reflected coefficient for the sea surface. Therefore, the ASF of the reflect AIS signal is:

(6) $$\alpha_{rASF}=kr_{1}+k\Delta r+\varphi_{\Gamma_{P}}$$

where [13]:

(7) $$\begin{array}{l} r_{1}=\sqrt{{\left(h_{t}-h_{r}\right)}^{2}+d^{2}}\\ r_{2}=\sqrt{{\left(h_{t}+h_{r}\right)}^{2}+d^{2}} \end{array}$$

where d is the propagation distance of the AIS signal. The reflection coefficient of the rough sea is the product of the smooth sea surface reflection coefficient, the diffusion factor decreasing the reflected coefficient due to the curvature of the Earth and the correction coefficient of the sea surface roughness [19], which is expressed as:

(8) $$\Gamma_{P}=\Gamma_{F}\times D_{f}\times \rho$$

In Equation (8), $\Gamma_{F}$ is the reflection coefficient for the smooth sea surface, which is Fresnel reflection coefficient.$D_{f}$ is the diffusion factor. ρ is the correction coefficient of the sea surface roughness. The Fresnel reflection coefficients of vertical polarization AIS signals given in [13] are:

(9) $$\begin{array}{ll} \Gamma_{V} & =\frac{1}{{\left(\varepsilon_{r}\sin\varphi +m\right)}^{2}+{\left(n-60\lambda \sigma \sin\varphi \right)}^{2}}\\ & \times [(\varepsilon_{r}^{2}+3600\lambda^{2}\sigma^{2})\sin^{2}\varphi -(m^{2}+n^{2})-j2\sin\varphi (n\varepsilon_{r}+60\lambda \sigma m)] \end{array}$$

m and n are expressed as [13]:

(10) $$\begin{array}{l} m=\sqrt{\frac{\varepsilon_{r}-\cos^{2}\varphi +\sqrt{{\left(\varepsilon_{r}-\cos^{2}\varphi \right)}^{2}+3600\lambda \sigma }}{2}}\\ n=-\frac{30\sqrt{2}\lambda \sigma }{\sqrt{\varepsilon_{r}-\cos^{2}\varphi +\sqrt{{\left(\varepsilon_{r}-\cos^{2}\varphi \right)}^{2}+3600\lambda^{2}\sigma^{2}}}} \end{array}$$

In Equation (10), λ is the wavelength of the AIS signal and φ is the grazing angle of the AIS signal. $\varepsilon_{r}$ and σ are relative permittivity and conductivity of seawater, respectively.

$D_{f}$ given in [18] is:

(11) $$D_{f}=\frac{1}{\sqrt{1+\frac{2r_{1}^{\prime }r_{2}^{\prime }}{R\left(r_{1}^{\prime }+r_{2}^{\prime }\right)\cos\theta_{i}}}}$$

where $\theta_{i}$ is the incident angle of the AIS signal. Miller and Brown proposed in [29] that the roughness correction factor of the sea surface could be expressed by Equation (11):

(12) $$\begin{array}{ll} \rho & =\exp\left[-2{\left(2\pi g\right)}^{2}\right]I_{0}\left[2\left(2\pi g\right)\right]\\ & =\exp\left[-2\left(2\pi \frac{\sigma_{h}\sin\varphi }{\lambda }\right)\right]I_{0}\left[2\left(2\pi \frac{\sigma_{h}\sin\varphi }{\lambda }\right)\right] \end{array}$$

In the formula, $\sigma_{h}$ is the standard deviation of the sea surface elevation. The standard deviation for the Phillips spectrum sea surface is a function of the wind speed on the sea surface, that is [30]:

(13) $$\sigma_{h}=0.0051U_{10}^{2}$$

In the formula, $U_{10}$ is the wind speed at 10 m above sea level. Considering Equations (6)–(13), it can be deduced that the ASFs of the AIS reflected signal is related to d, $\varepsilon_{r}$, *σ*, and $U_{10}$ while the calculation of $\varepsilon_{r}$ and *σ* refers to the Debye formulas [31]. The relative permittivity and conductivity of seawater given in the Debye formulas are related to seawater temperature and salinity. Therefore, the ASFs of the AIS reflected signal changes with d, T, S, and $U_{10}$.

Assuming that the transmitting antenna $h_{t}$ is 50 m and the receiving antenna $h_{r}$ is 20 m, taking into account the refraction effect of the atmosphere on the ultrashort wave, the line-of-sight propagation distance is corrected and expressed as [14]:

(14) $$r_{0}=4.12\left(\sqrt{h_{t}}\left(m\right)+\sqrt{h_{r}}\left(m\right)\right)\left(\mathrm{km}\right)$$

**Figure 2:**
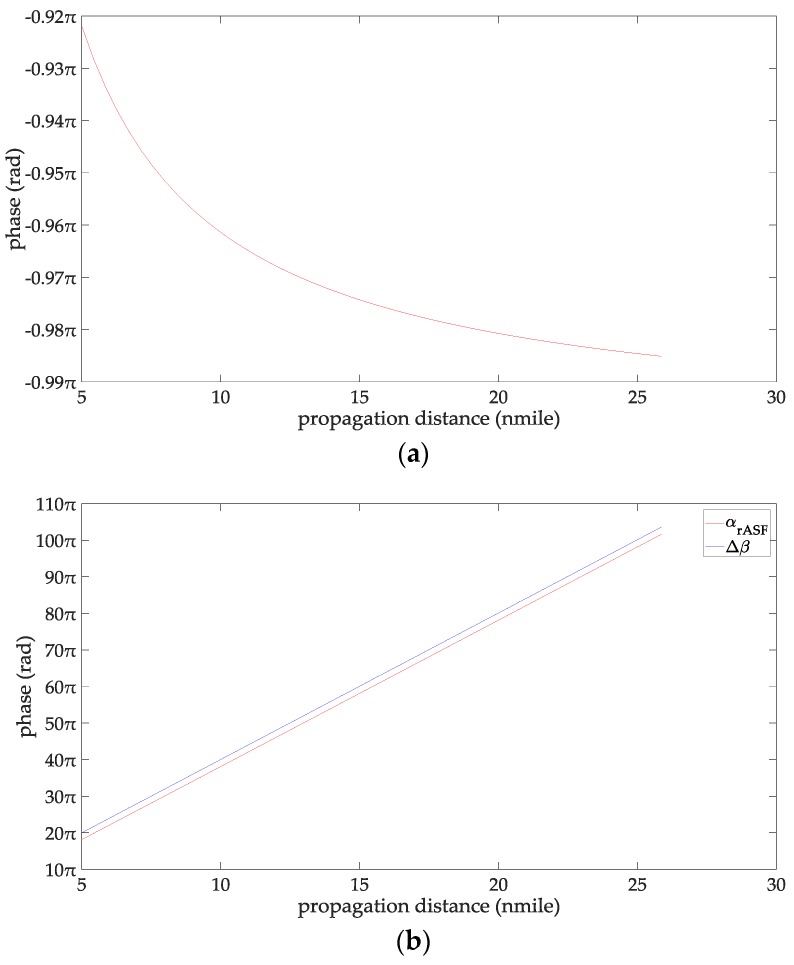
(**a**) The phases of the reflection coefficient changing with the propagation distance; and (**b**) the ASFs of the AIS reflected signal changing with the propagation distance.

**Figure 3:**
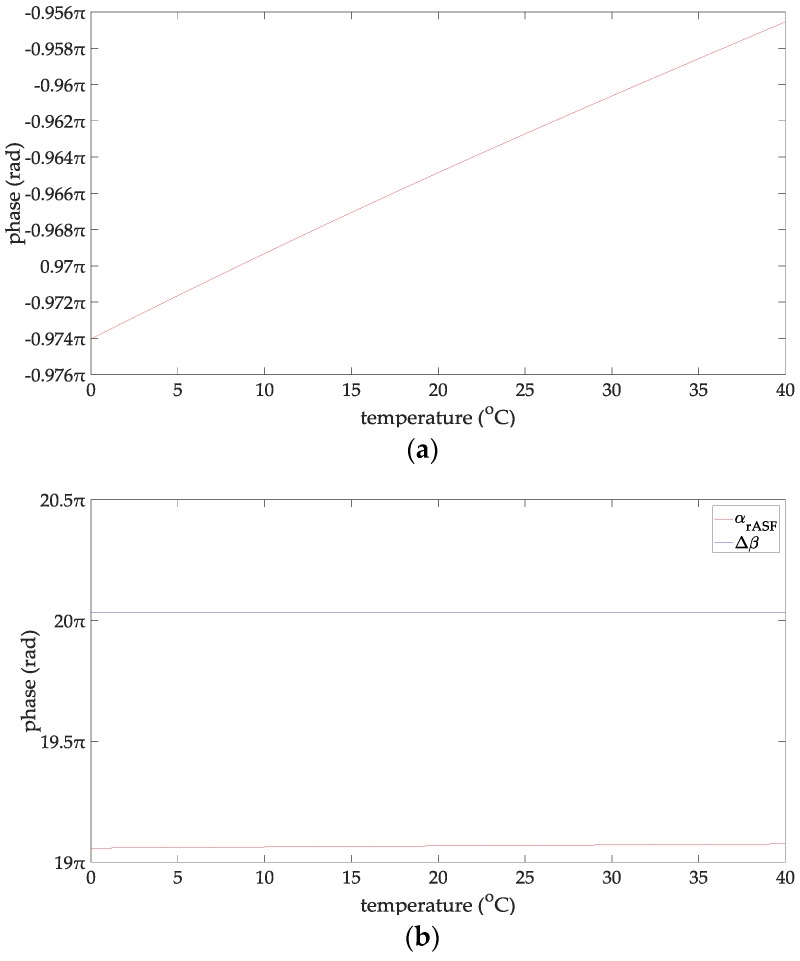
(**a**) The phases of the reflection coefficient changing with the sea temperature; and (**b**) the ASFs of the AIS reflected signal changing with the sea temperature.

**Figure 4:**
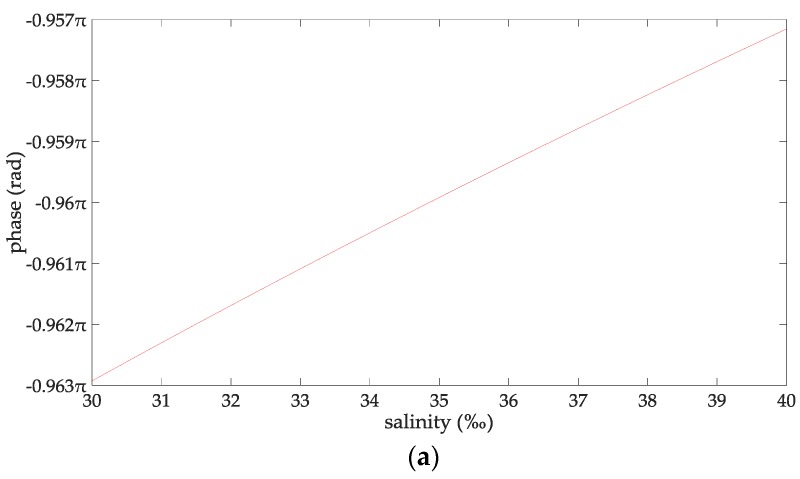

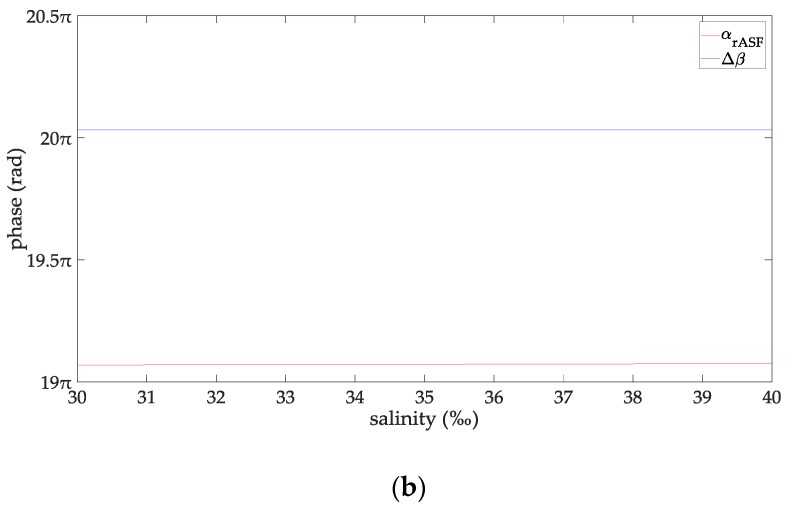
(**a**) The phases of the reflection coefficient changing with the sea salinity; and (**b**) the ASFs of the AIS reflected signal changing with the sea salinity.

**Figure 5:**
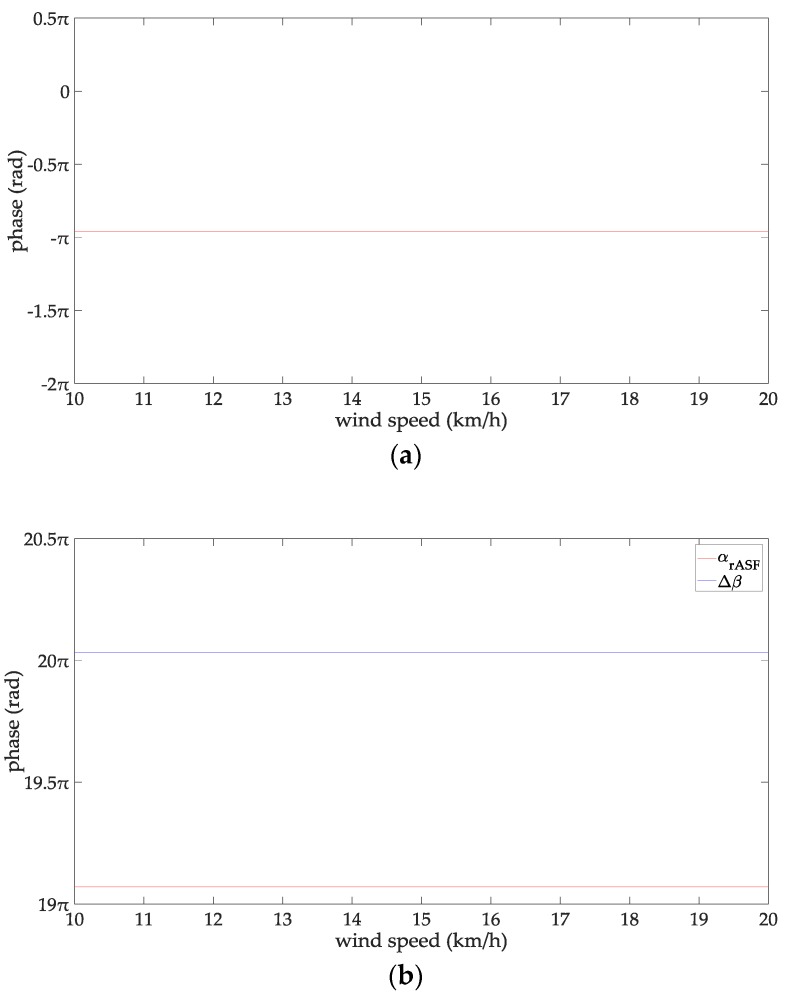
(**a**) The phases of the reflection coefficient changing with the wind speed; and (**b**) the ASFs of the AIS reflected signal changing with the wind speed.

At this time, the line-of-sight propagation distance is 47 km, which is about 25 nautical miles. Therefore, the propagation distance of the AIS signal studied in this paper is 5–25 n miles. Since there is no clear standard for the definition of the wave height for a rough sea surface, this paper chooses empirical data by referring to the relevant contributions and their experimental conditions. In particular [32,33,34] gave relatively consistently reference data. From [32,33,34], when the wind speed is 38–78 km/h, the sea surface could be considered as be rough for the radio wave frequency of 1–170 MHz. According to the wind scale classification in the Beaufort scale, the wave height is 2–7 m at this time. Therefore, the range of the wind speed concerned in this paper is 38–78 km/h, and the wave height is 2–7 m. When the seawater temperature is 28 °C, the seawater salinity is 32.54‰ and the wind speed is 54 km/h, and the changing trend charts shows the phases of the reflection coefficient for the rough sea surface and the ASFs of the AIS reflected signal in Figure 2. When the sea salinity is 32.54‰, the propagation distance is 10 nautical miles, the wind speed is 54 km/h, and the changing trend charts shows the phases of the reflection coefficient for the rough sea surface and the ASFs of the AIS reflected signal in Figure 3. When the sea temperature is 28 °C, the propagation distance is 10 nautical miles and the wind speed is 54 km/h, and the changing trend charts shows the phases of the reflection coefficient for the rough sea surface and the ASFs of the AIS reflected signal in Figure 4. When the sea temperature is 28 °C, the sea salinity is 32.54‰, the propagation distance is 10 nautical miles, the changing trend charts shows the phases of the reflection coefficient for the rough sea surface and the ASFs of the AIS reflected signal in Figure 5.

According to Figure 2, the phase of the reflection coefficient for an AIS signal is −0.985 to −0.922π, the ASF of the AIS reflected signal is 9.09–50.81π. The phase of the AIS direct signal is 10.016–51.79π. Within the propagation distance range of 5–25 n miles, it can be deduced that the phases of the reflection coefficient decrease with increasing the propagation distance, their variation is 0.063 π. The phases of the AIS direct signal, the ASFs of the AIS reflected signal increase with increasing the propagation distance, their variations are 41.72π, and 41.76 π, respectively. According to Figure 3, the phase of the reflection coefficient for an AIS signal is –0.974 to –0.957π, the ASF of the AIS reflected signal is 19.058–19.075π. The phase of the AIS direct signal is 20.032π. It can be deduced that within the seawater temperature of 0–40 °C, the phases of the reflection coefficient and the ASFs of the AIS reflected signal increase with increase in the seawater temperature; their variations are 0.017π. The phase of the AIS direct signal is a constant when the seawater temperature is 0–40 °C. According to Figure 4, the phase of the reflection coefficient is −0.963 to −0.957π; the ASF of the AIS reflected signal is 19. 069–19.075π. The phase of the AIS direct signal is 20.032π. Within the sea salinity range of 30–40‰, it can be deduced that the phases of the reflection coefficient and the ASFs of the AIS reflected signal increase with increasing the sea salinity; their variations are 0.006π, which could be regarded as constants. According to Figure 5, the phase of the reflection coefficient is −0.964–−0.963π, the ASF of the AIS reflected signal is 19.068–19.069π. The phase of the AIS direct signal is 20.032π. It can be deduced that within the wind speed range of 38–78 km/h. the phases of the reflection coefficient and the ASFs of the AIS reflected signal decrease with the increase of the wind speed; their variations are 0.001π, which could be regarded as constants. In summary, the ASFs of the AIS reflected signal is most affected by the propagation distance. The sea salinity and the wind speed have no impact on the ASFs of the AIS reflected signal for vertical polarization AIS signal. The seawater temperature has a slight effect on the ASFs of the AIS reflected signal. However, if the AIS signal is to be used for highly-accurate range positioning, the influence of this factor on the ASFs should be considered in order to obtain smaller positioning errors.

## 3. The ASFs of the AIS Diffracted Signal

When the sea surface is rough, the rolling waves will block the AIS signal propagation, and the AIS signal will continue to spread around the waves in a diffractive way [35]. Diffraction will cause a significant energy attenuation of the received signal, distort the phases of the received signal, and reduce the communication quality [28]. It could be seen that the diffraction phenomenon is also one of the factors that affects the AIS received signal. Since this paper is aimed at the ASF correction of AIS received signals under severe weather conditions, it is necessary to study on the diffraction phenomenon of AIS signals. This section will study the relationship among the wave heights, the clearance parameters of the wave and the ASFs of the AIS diffracted signal transmitted on the rough sea surface and give the changing trend charts and numerical results of the ASFs for the AIS diffracted signal.

### 3.1. The Clearance Parameters of the Wave

**Figure 6:**
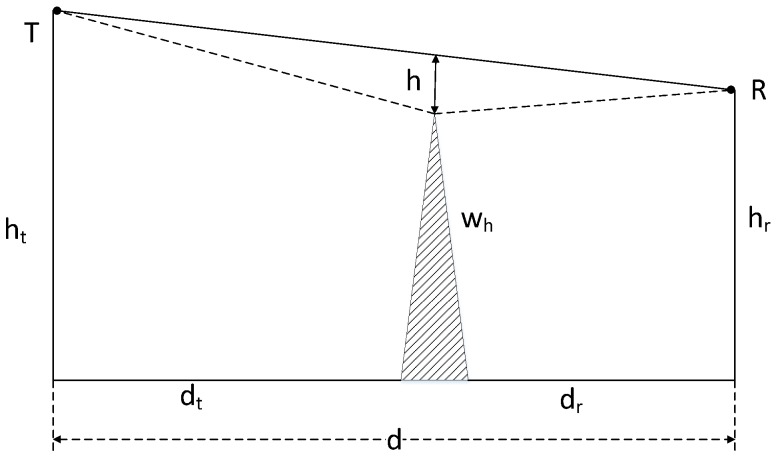
The diagram of the clearance parameter.

Considering [36] concluded that the propagation loss and field strength of the diffracted signal are both related to the clearance parameter, so this section gives the clearance parameter of the wave in the AIS direct signal path, as shown in Figure 6.

In Figure 6, *T*, *R* are the transmitted and received points respectively. $h_{t}$ is the height of the transmitted antenna and $h_{r}$ is the height of the received antenna. $d_{t}$ is the horizontal distance from the wave to the transmitted point. $d_{r}$ is the horizontal distance from the wave to the received point. d is the propagation distance of the AIS signal, where $d_{r}=d-d_{t}$. $w_{h}$ is the height of the wave and h is the clearance parameter of the wave. According to Figure 6:

(15) $$\frac{\left(w_{h}+h\right)}{h_{t}}=\frac{d_{r}}{d_{t}}$$

Therefore, h could be expressed as:

(16) $$h=\frac{h_{t}\left(d-d_{t}\right)}{d_{t}}-w_{h}$$

In Equation (16), the units of h, d, $d_{t}$, $h_{t}$ are m.

### 3.2. The ASFs of the AIS Diffracted Signal over the Rough Sea Surface

Reference [34] regarded the wave on the rough sea surface as a knife-edge obstacle, so this section also uses the diffraction method of a knife-edge obstacle diffraction method to calculate the field strength of the received point obtain the ASFs of the AIS diffracted signal on the rough sea surface, which is [37]:

(17) $$E_{d}=E_{0}\frac{\exp\left(-jkd\right)}{d}\times \sqrt{\frac{j}{2}}\times \int_{v_{0}}^{\infty }\exp\left(-\frac{j\pi }{2}v^{2}\right)dv$$

where k is the wave number of the AIS signal. d is the propagation distance, $v_{0}=\sqrt{2}\frac{h}{F_{1}}$, and $F_{1}$ is the first Fresnel radius of the obstacle point. According to [38], $F_{1}$ is:

(18) $$F_{1}=\sqrt{\frac{\lambda d_{t}d_{r}}{\left(d_{t}+d_{r}\right)}}$$

The integral item of Equation (17) can be expressed as:

(19) $$\begin{array}{ll} \int_{v_{0}}^{\infty }\exp(-\frac{j\pi }{2}v^{2})dv & =\int_{0}^{\infty }\exp(-\frac{j\pi }{2}v^{2})dv-\int_{0}^{v_{0}}\exp(-\frac{j\pi }{2}v^{2})dv\\ & =C(\infty )-jS(\infty )-[C(v_{0})-jS(v_{0})]\\ & =\frac{1}{2}\left(1-j\right)-\left[C(v_{0})-jS(v_{0})\right] \end{array}$$

where $C\left(v_{0}\right)$ and $S\left(v_{0}\right)$ are Fresnel integral terms, which are denoted as [26]:

(20) $$\begin{array}{l} C\left(v_{0}\right)=\int_{0}^{v_{0}}\cos\left(\frac{\pi }{2}v^{2}\right)dv\\ S\left(v_{0}\right)=\int_{0}^{v_{0}}\sin\left(\frac{\pi }{2}v^{2}\right)dv \end{array}$$

Substituting Equation (19) into Equation (17) we obtain:

(21) $$E_{d}=E_{0}\frac{e^{j\left(kd-\frac{\pi }{4}\right)}}{\sqrt{2}d}\left\{\frac{1}{2}\left(1-j\right)-\left[C\left(v_{0}\right)-jS\left(v_{0}\right)\right]\right\}$$

ITU-R P.526-12 Recommendation [26] states that a sufficiently accurate Fresnel integral is provided by applying Boersma coefficients in most cases, namely:

(22) $$F_{c}(v)=\exp(jx)\sqrt{\frac{x}{4}}\sum_{n=0}^{11}\left[(a_{n}-jb_{n}){\left(\frac{x}{4}\right)}^{n}\right]\;0\leq x\leq 4$$

(23) $$F_{c}(v)=\left(\frac{1+j}{2}\right)+\exp(jx)+\sqrt{\frac{4}{x}}\sum_{n=0}^{11}\left[\left(c_{n}-jd_{n}\right){\left(\frac{4}{x}\right)}^{n}\right]\;x>4$$

where $x=\frac{\pi }{2}v^{2}$. Assuming that the height of the transmitting antenna is 50 m, the height of the received antenna is 20 m, the propagation distance of the AIS signal is 10 nautical miles, and the wave height is 2–7 m. Equation (24) is derived by calculating and deducing Equations (22) and (23):

(24) $$\begin{array}{ll} \int_{v_{0}}^{\infty }\exp(-\frac{j\pi }{2}v^{2})dv & =\frac{1}{2}\left(1-j\right)-\left[C(v_{0})-jS(v_{0})\right]\\ & =\frac{1}{2}\left(1-j\right)-\exp(jx)\sqrt{\frac{x}{4}}\sum_{n=0}^{11}\left[(a_{n}+jb_{n}){\left(\frac{x}{4}\right)}^{n}\right],0\leq x<4\\ & =-\exp(jx)\sqrt{\frac{4}{x}}\left[\sum_{n=0}^{11}\left(c_{n}+jd_{n}\right){\left(\frac{4}{x}\right)}^{n}\right],x>4 \end{array}$$

The Taylor series expansion is carried out for $e^{jx}$ in Equation (24). Since the *x*-value in $e^{jx}$ ranges from 0 to π, it needs to perform some processing for cases when *x* is greater than π in Equation (24). When *x* is greater than π, *p* is the integer part of $\frac{x}{\pi }$, set t=x−pπ, the Taylor series expansion of $e^{jt}$ is performed for this *t* value. The Taylor series expansion value of $e^{jt}$ is equal to $e^{j\left(t+p\pi \right)}$, so t can substitute *x* for the Taylor series expansion of $e^{jx}$. For the case that *x* is smaller than π, Taylor series expansion can be performed directly without much processing. It is obtained after many derivation calculations that, when $e^{jx}$ is expanded to the thirteenth power item, the degree of fitting is the optimal between the $e^{jx}$ phase expanded by Taylor series and the phase directly calculated by Matlab.

**Figure 7:**
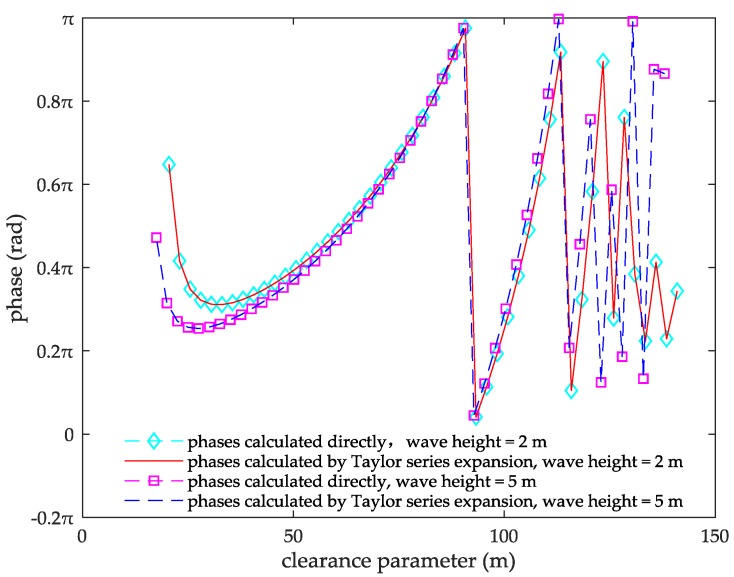
The fitting chart of phases calculated directly and the Taylor series expansion.

From Figure 7, when $e^{jx}$ is expanded to the thirteenth power item, the phase expanded by Taylor series is completely fitted to the phase calculated directly by Matlab. The relationship between the degree of fitting and the numbers of the Taylor series expansion items is not affected by the wind speed. $e^{jx}$ can be expanded to the thirteenth power item of Taylor series, regardless of the wind speed. The Taylor series expansion of $e^{jx}$ is:

(25) $$\left\{ \begin{array}{l} e^{jx}=\sum_{m=0}^{6}{\left(-1\right)}^{m}\frac{x^{2m}}{(2m)!}+j\sum_{m=0}^{6}{\left(-1\right)}^{m}\frac{x^{2m+1}}{(2m+1)!},0\leq x\leq \pi \\ e^{jt}=\sum_{m=0}^{6}{\left(-1\right)}^{m}\frac{t^{2m}}{(2m)!}+j\sum_{m=0}^{6}{\left(-1\right)}^{m}\frac{t^{2m+1}}{(2m+1)!},t=x-p\pi ,x>\pi \end{array}\right.$$

Therefore, the ASFs of the AIS diffracted signal over a rough sea surface,

(26) $$\alpha_{dASF}=kd-\frac{\pi }{4}+\arctan\left\{\begin{array}{l} \frac{-\frac{1}{2}-\sqrt{\frac{x}{4}}\left\{\sum_{n=0}^{11}a_{n}{\left(\frac{x}{4}\right)}^{n}\left[\sum_{m=0}^{6}{\left(-1\right)}^{m}\frac{x^{2m+1}}{(2m+1)!}\right]-\sum_{n=0}^{11}b_{n}{\left(\frac{x}{4}\right)}^{n}\left[\sum_{m=0}^{6}{\left(-1\right)}^{m}\frac{x^{2m}}{(2m)!}\right]\right\}}{\frac{1}{2}-\sqrt{\frac{x}{4}}\left\{\sum_{n=0}^{11}a_{n}{\left(\frac{x}{4}\right)}^{n}\left[\sum_{m=0}^{6}{\left(-1\right)}^{m}\frac{x^{2m}}{(2m)!}\right]+\sum_{n=0}^{11}b_{n}{\left(\frac{x}{4}\right)}^{n}\left[\sum_{m=0}^{6}{\left(-1\right)}^{m}\frac{x^{2m+1}}{(2m+1)!}\right]\right\}},0\leq x\leq \pi \\ \frac{-\frac{1}{2}-\sqrt{\frac{x}{4}}\left\{\sum_{n=0}^{11}a_{n}{\left(\frac{x}{4}\right)}^{n}\left[\sum_{m=0}^{6}{\left(-1\right)}^{m}\frac{t^{2m+1}}{(2m+1)!}\right]-\sum_{n=0}^{11}b_{n}{\left(\frac{x}{4}\right)}^{n}\left[\sum_{m=0}^{6}{\left(-1\right)}^{m}\frac{t^{2m}}{(2m)!}\right]\right\}}{\frac{1}{2}-\sqrt{\frac{x}{4}}\left\{\sum_{n=0}^{11}a_{n}{\left(\frac{x}{4}\right)}^{n}\left[\sum_{m=0}^{6}{\left(-1\right)}^{m}\frac{t^{2m}}{(2m)!}\right]+\sum_{n=0}^{11}b_{n}{\left(\frac{x}{4}\right)}^{n}\left[\sum_{m=0}^{6}{\left(-1\right)}^{m}\frac{t^{2m+1}}{(2m+1)!}\right]\right\}}t=x-p\pi ,\pi <x\leq 4\\ \frac{-\sum_{m=0}^{6}{\left(-1\right)}^{m}\frac{t^{2m+1}}{(2m+1)!}\sqrt{\frac{4}{x}}\sum_{n=0}^{11}\left(c_{n}\right){\left(\frac{4}{x}\right)}^{n}-\sum_{m=0}^{6}{\left(-1\right)}^{m}\frac{t^{2m}}{(2m)!}\sum_{n=0}^{11}\left(d_{n}\right){\left(\frac{4}{x}\right)}^{n}}{\sum_{m=0}^{6}{\left(-1\right)}^{m}\frac{t^{2m+1}}{(2m+1)!}\sum_{n=0}^{11}\left(d_{n}\right){\left(\frac{4}{x}\right)}^{n}-\sum_{m=0}^{6}{\left(-1\right)}^{m}\frac{t^{2m}}{(2m)!}\sum_{n=0}^{11}\left(c_{n}\right){\left(\frac{4}{x}\right)}^{n}}t=x-p\pi ,x>4 \end{array}\right\}$$

The probability density function of the wave is [29]:

(27) $$\begin{array}{ll} D\left(y\right) & =\frac{1}{\pi^{3/2}\sigma_{h}}\int_{y}^{\infty }\frac{\exp\left(-\frac{H^{2}}{4\sigma_{h}}\right)}{\sqrt{H^{2}-y^{2}}}dH\\ & =\frac{1}{2\pi^{3/2}\sigma_{h}}\exp\left(-\frac{y^{2}}{8\sigma_{h}}\right)K_{0}\left(\frac{y^{2}}{8\sigma_{h}}\right) \end{array}$$

**Figure 8:**
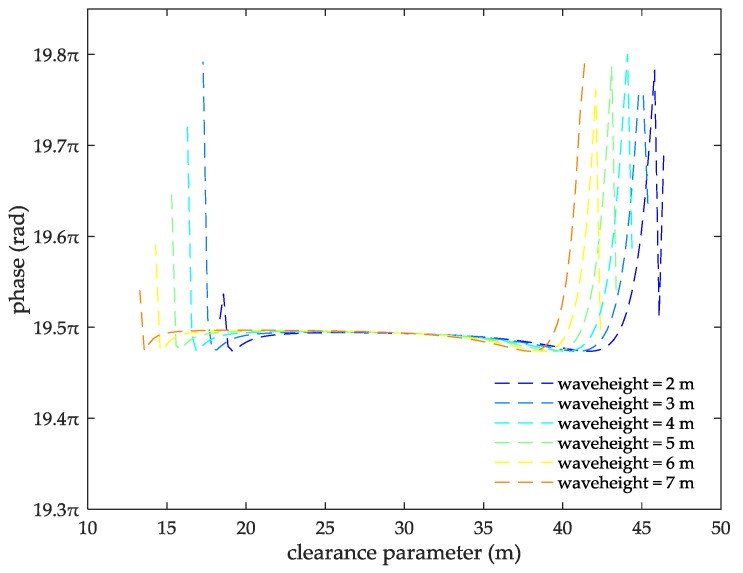
The ASFs of the AIS diffracted signal changing with the clearance parameters for different wave heights.

In Equation (27), $\sigma_{h}$ is the standard deviation of the sea surface elevation calculated by Equation (13). As the AIS signal is diffracted at the top of the ocean wave the clearance parameters for different wave heights are calculated by Equation (16). Then, the ASFs of the AIS diffracted signal generated by different clearance parameters are calculated by Equation (26) as shown in Figure 8. The data of Figure 8 are summarized in Table 1.

From the information in Table 1, it can be seen that clearance parameter of the AIS signal changes with the position of the sea-wave from the transmitted point, and the closer the diffracted point to the transmitted point, the larger the clearance parameter when the sea temperature is 28 °C, the propagation distance is 10 nautical miles and the wave height is 2–7 m. The clearance parameter of the AIS diffracted signal varies with the wave height. At the same sea-wave position, the higher the wave height, the smaller the clearance parameter. The ASFs of the AIS diffracted signal show a trajectory of decrease first, increase then, decrease again and increase then with the increase of the clearance parameters. However, as for the wave height of 2 m, there will be a decreasing and then increasing oscillation after the same trend as the other wave heights. At the same clearance parameter, the ASFs of the AIS diffracted signal decreases with the increase of the wave height. For example, when the clearance parameter is 34 m, the ASFs of the AIS diffracted signal generated at a wave height of 3 m is higher than the ASFs at a wave height of 5 m. For different wave heights, the ASFs of the AIS diffracted signal feature same changing trend. They have basically same maximum value and minimum value. The maximum ASF of the AIS diffracted signal over a rough sea surface appear at the maximum value point and minimum value point of the clearance parameter.

## 4. Results and Discussion

### 4.1. The ASFs of the AIS Received Signal

The previous two sections mainly studied the relationship between the ASFs of the AIS reflected signal and diffracted signal and their influence factors and gave the calculated numerical values of the ASFs under certain conditions. The superimposition ASFs of the reflected signal and diffracted signal could be considered as the ASFs of the AIS received signal, that is:

(28) $$\begin{array}{ll} \alpha_{ASF} & =\alpha_{rASF}+\alpha_{dASF}\\ & =\frac{2\pi }{\lambda }r_{1}+k\Delta r+\varphi_{\Gamma_{P}}+kd-\frac{\pi }{4}+\arctan\left\{\begin{array}{l} \frac{-\frac{1}{2}-\sqrt{\frac{x}{4}}\left\{\sum_{n=0}^{11}a_{n}{\left(\frac{x}{4}\right)}^{n}\left[\sum_{m=0}^{6}{\left(-1\right)}^{m}\frac{x^{2m+1}}{(2m+1)!}\right]-\sum_{n=0}^{11}b_{n}{\left(\frac{x}{4}\right)}^{n}\left[\sum_{m=0}^{6}{\left(-1\right)}^{m}\frac{x^{2m}}{(2m)!}\right]\right\}}{\frac{1}{2}-\sqrt{\frac{x}{4}}\left\{\sum_{n=0}^{11}a_{n}{\left(\frac{x}{4}\right)}^{n}\left[\sum_{m=0}^{6}{\left(-1\right)}^{m}\frac{x^{2m}}{(2m)!}\right]+\sum_{n=0}^{11}b_{n}{\left(\frac{x}{4}\right)}^{n}\left[\sum_{m=0}^{6}{\left(-1\right)}^{m}\frac{x^{2m+1}}{(2m+1)!}\right]\right\}},0\leq x\leq \pi \\ \frac{-\frac{1}{2}-\sqrt{\frac{x}{4}}\left\{\sum_{n=0}^{11}a_{n}{\left(\frac{x}{4}\right)}^{n}\left[\sum_{m=0}^{6}{\left(-1\right)}^{m}\frac{t^{2m+1}}{(2m+1)!}\right]-\sum_{n=0}^{11}b_{n}{\left(\frac{x}{4}\right)}^{n}\left[\sum_{m=0}^{6}{\left(-1\right)}^{m}\frac{t^{2m}}{(2m)!}\right]\right\}}{\frac{1}{2}-\sqrt{\frac{x}{4}}\left\{\sum_{n=0}^{11}a_{n}{\left(\frac{x}{4}\right)}^{n}\left[\sum_{m=0}^{6}{\left(-1\right)}^{m}\frac{t^{2m}}{(2m)!}\right]+\sum_{n=0}^{11}b_{n}{\left(\frac{x}{4}\right)}^{n}\left[\sum_{m=0}^{6}{\left(-1\right)}^{m}\frac{t^{2m+1}}{(2m+1)!}\right]\right\}}t=x-p\pi ,\pi <x\leq 4\\ \frac{-\sum_{m=0}^{6}{\left(-1\right)}^{m}\frac{t^{2m+1}}{(2m+1)!}\sqrt{\frac{4}{x}}\sum_{n=0}^{11}\left(c_{n}\right){\left(\frac{4}{x}\right)}^{n}-\sum_{m=0}^{6}{\left(-1\right)}^{m}\frac{t^{2m}}{(2m)!}\sum_{n=0}^{11}\left(d_{n}\right){\left(\frac{4}{x}\right)}^{n}}{\sum_{m=0}^{6}{\left(-1\right)}^{m}\frac{t^{2m+1}}{(2m+1)!}\sum_{n=0}^{11}\left(d_{n}\right){\left(\frac{4}{x}\right)}^{n}-\sum_{m=0}^{6}{\left(-1\right)}^{m}\frac{t^{2m}}{(2m)!}\sum_{n=0}^{11}\left(c_{n}\right){\left(\frac{4}{x}\right)}^{n}}t=x-p\pi ,x>4 \end{array}\right\} \end{array}$$

**Figure 9:**
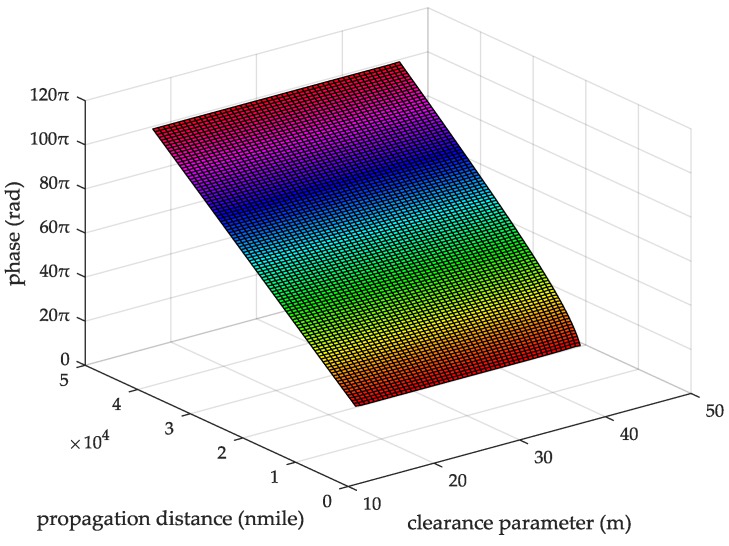
The ASFs of the AIS received signal changing with the propagation distance and clearance parameter.

**Figure 10:**
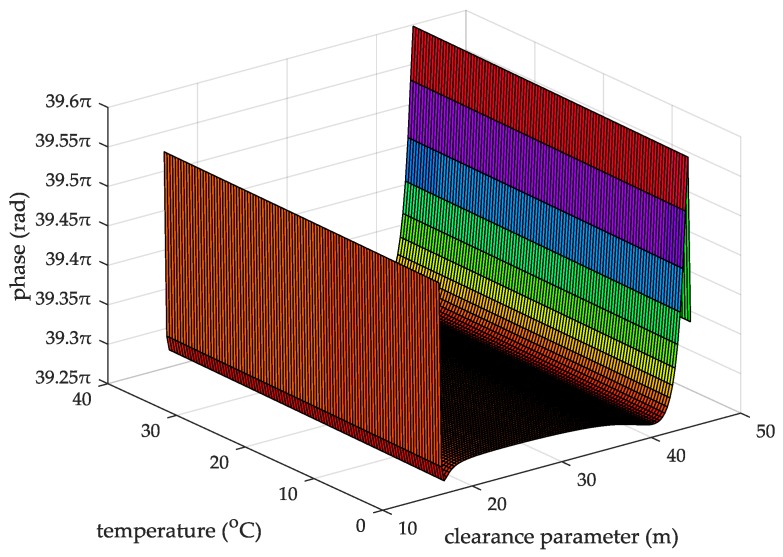
The ASFs of the AIS received signal changing with the seawater temperature and the clearance parameter.

**Figure 11:**
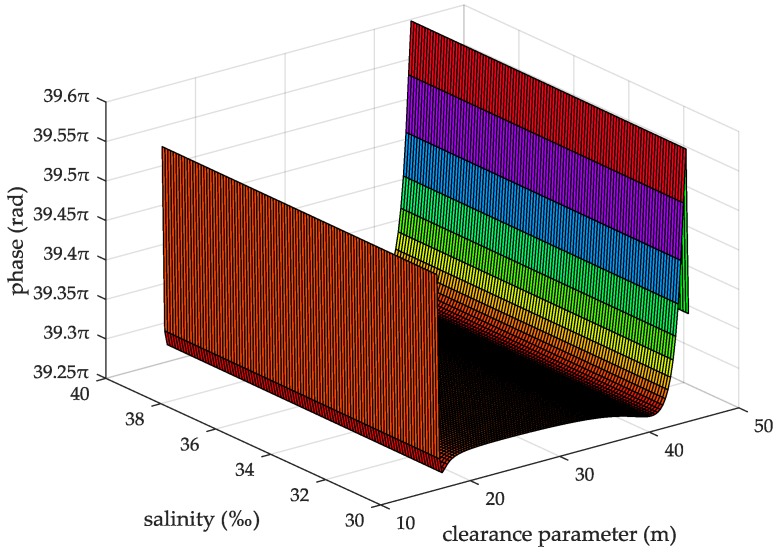
The ASFs of the AIS received signal changing with the sea salinity and the clearance parameter.

**Figure 12:**
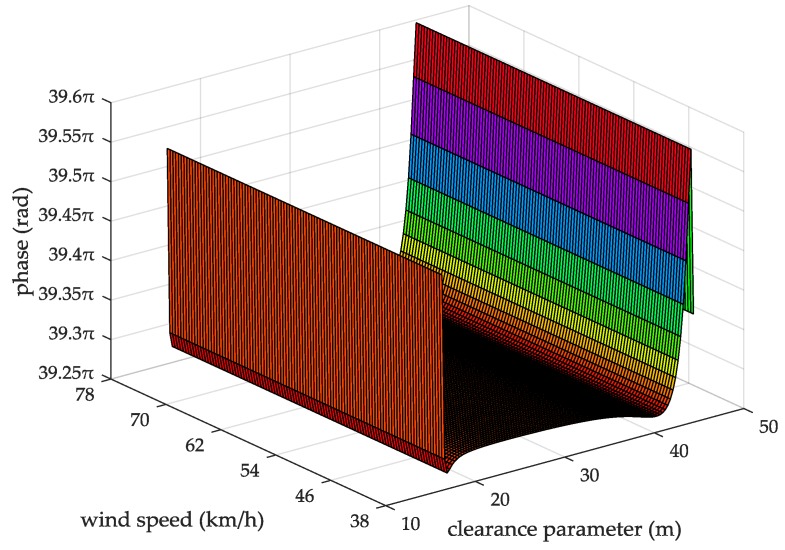
The ASFs of the AIS received signal changing with the wind speed and the clearance parameter.

**Figure 13:**
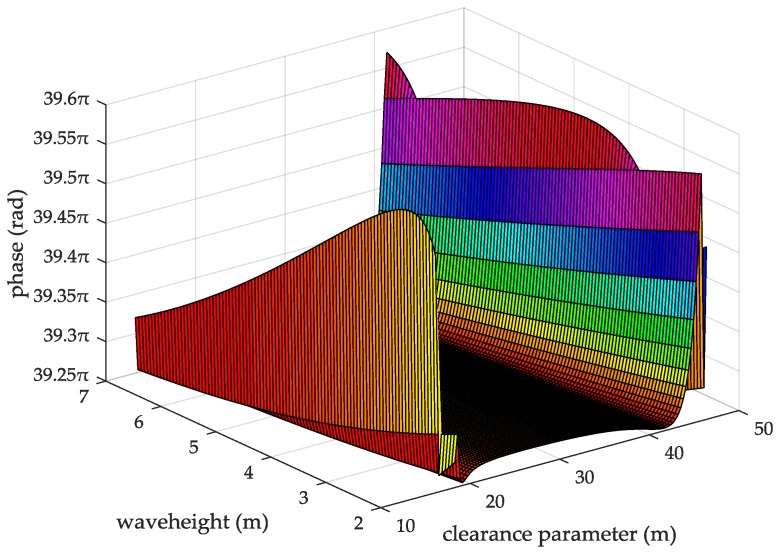
The ASFs of the AIS received signal changing with the wave heights and the clearance parameters.

When the seawater temperature is 28 °C, the sea salinity is 32.54‰, the wind speed is 54 km/h, the average wave height is 4 m, and the changing trend chart shows the ASFs of the AIS received signal varying with the propagation and the clearance parameter in Figure 9. When the sea salinity is 32.54‰, the propagation distance is 10 nautical miles, the wind speed is 54 km/h, the average wave height is 4 m, and the changing trend chart shows that the ASFs of the AIS received signal vary with the seawater temperature and the clearance parameter in Figure 10. When the seawater temperature is 28 °C, the propagation distance is 10 nautical miles, the wind speed is 54 km/h, the average wave height is 4 m, and the changing trend chart shows that the ASFs of the AIS received signal vary with the sea salinity and the clearance parameter in Figure 11. When the seawater temperature is 28 °C, the sea salinity is 32.54‰, the propagation distance is 10 nautical miles, the average wave height is 4 m, and the changing trend chart shows the ASFs of the AIS received signal varying with the wind speed and clearance parameter in Figure 12. When the seawater temperature is 28 °C, the sea salinity is 32.54‰, the propagation distance is 10 nautical miles, the wind speed is 54 km/h, and the changing trend charts show that the ASFs of the AIS received signal varying with the wave height and the clearance parameter in Figure 13.

From the information in Figure 9, when the seawater temperature is 28 °C, the sea salinity is 32.54‰, the wind speed is 54 km/h, the average wave height is 4 m, within the propagation distance range of 5–25 nautical miles, the clearance parameter is 16–45.38 m and the ASF of the AIS received signal is 19.34–102.64π. In this case, the range positioning error corrections of the AIS signal is 1.79–9.51 m.

From the information in Figure 10, when the sea salinity is 32.54‰, the propagation distance is 10 nautical miles, the wind speed is 54 km/h, the average wave height is 4 m, within the seawater temperature range of 0–40 °C, the clearance parameter is 16–44.38 m and the ASF of the AIS received signal is 39.27–39.6π. In this case, the range positioning error corrections of the AIS received signal is 3.64–3.67 m.

From the information in Figure 11, when the seawater temperature is 28 °C, the propagation distance is 10 nautical miles, the wind speed is 54 km/h, the average wave height is 4 m, the sea salinity is 30–40‰, the clearance parameter is 16–44.38 m and the ASF of the AIS received signal is 39.27–39.6π. In this case, the range positioning error corrections of the AIS received signal is 3.64–3.67 m. From the information in Figure 12, when the seawater temperature is 28 °C, the sea salinity is 32.54‰, the propagation distance is 10 nautical miles, the average wave height is 4 m, the wind speed is 38–78 km/h, the clearance parameter is 16–44.38 m and the ASF of the AIS received signal is 39.27–39.6π. In this case, the range positioning error corrections of the AIS signal is 3.64–3.67 m.

From the information in Figure 13, when the seawater temperature is 28 °C, the sea salinity is 32.54‰, the propagation distance is 10 nautical miles, the wind speed is 54 km/h, within the wave height is 2–7 m, the clearance parameter is 13–46.38 m and the ASFs of the AIS received signal is 39.2–39.53π. In this case, the range positioning error corrections of the AIS signal is 3.63–3.66 m. Thus, it can be seen that the ASFs and the range positioning error corrections of the AIS received signal increase with increasing the propagation distance. For the same clearance parameter, the ASFs and the range positioning error corrections of the AIS received signal increase with increases in the seawater temperature, the sea salinity, the wind speed, and the wave height. For the same seawater temperature, the ASFs and the range positioning error corrections of the AIS received signal present twice-upward trend with increasing the clearance parameter. Similar trends also present at the same sea salinity, wind speed and wave height points.

### 4.2. The Application of the ASFs in the AIS Positioning System

**Figure 14:**
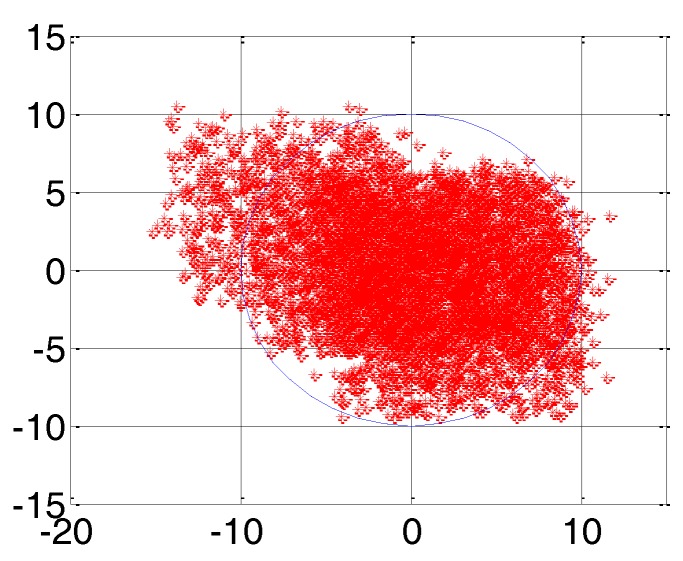
The ASFs of the AIS received signal correcting the positioning error.

First of all, a large-scale positioning error correction is performed on the AIS land-based positioning system by using the additional secondary phase correction system developed in [17], namely “coarse tuning” to achieve its positioning error within 10 m (2*σ*) when GDOP is less than 1.5. Then a small-scale positioning error correction is performed on the positioning system after “coarse tuning” by using the ASFs of the AIS received signal obtained in this paper, that is, “fine adjustment” to further correct the positioning error of the AIS. The authors apply the ASFs of the AIS received signal to data from the static positioning experiment in [17] to verify the effect of positioning error correction. Since the authors did not encounter the extreme weather of a violent storm in the authors’ city waters at that time, the complete result of the ASFs could be used. Based on the sea conditions at that time, the wave heights did not cause the diffraction of the AIS signal in the propagation path. Therefore, the authors removed the ASFs of the AIS diffracted signal from the ASFs of the AIS received signal and only the ASFs of the AIS reflected signal remained to correct the positioning error of the static positioning experimental data. The positioning error after ASF correction is shown in Figure 14. The longitude and latitude errors are represented by the horizontal and vertical axis, respectively. The mean and the root mean square (RMS) of the longitude error were −0.311 m and 2.57, respectively. The mean of the latitude error was 0.807 m and the RMS was 2.563 m. There were 5423 valid data points collected, and there were 5260 data points in the blue circle. The radius of the blue circle was 10 m and only 3% of all the positioning results were not in this positioning error circle. Therefore, the positioning error for the position was 7.259 m (2*σ*) after ASF correction.

### 4.3. Discussion

In this section, when the seawater temperature is 28 °C, the sea salinity is 32.54‰, the wind speed is 54 km/h, the average wave height is 4 m, within the propagation distance range of 5–25 nautical miles, the ASF of the AIS received signal is 19–102.64π, and the range positioning error correction is 1.79–9.51 m. When the propagation distance is 10 nautical miles, the seawater temperature is 28 °C, the wind speed is 54 km/h, and the average wave height is 4 m, within the sea salinity range of 30–40‰, the ASF of the AIS received signal is 39.26–39.6π, the range positioning error correction is 3.64–3.67 m. When the sea salinity is 32.54‰, the propagation distance is 10 nautical miles, the wind speed is 54 km/h, the average wave height is 4 m, within the seawater temperature range of 0–40 °C, the ASF of the AIS received signal is 39.27–39.6π, and the range positioning error correction is 3.64–3.67 m. When the seawater temperature is 28 °C, the sea salinity is 32.54‰, the propagation distance is 10 nautical miles, the average wave height is 4 m, within the wind speed range of 38–78 km/h, the ASF of the AIS received signal is 39.27–39.6π, and the range positioning error correction is 3.64–3.67 m. When the seawater temperature is 28 °C, the sea salinity is 32.54‰, the wind speed is 54 km/h, the propagation distance is 10 nautical miles, within the wave height range of 2–7 m, the clearance parameter of the AIS signal is 13–46.39 m, the ASF of the AIS received signal is 39.2–39.53π, the range positioning error correction is 3.63–3.66 m. Thus, it can be seen that the ASFs of the AIS received signal and the range positioning error correction increase with increasing propagation distance. For the same clearance parameter, the ASFs and the range positioning error corrections of the AIS received signal increase with increases in the seawater temperature, the sea salinity, the wind speed, and the wave height. For the same seawater temperature, the ASFs and the range positioning error corrections of the AIS received signal present twice-upward trend with increasing the clearance parameter. Similar trends also present at the same sea salinity, wind speed and wave height points. The range positioning error corrections obtained by the experiment were sent to the additional secondary phase correction system for AIS signals developed in [17] and after “coarse tuning”; the positioning error was “fine-tuned” by the additional secondary phase correction system to obtain more accurate positioning data. By further correcting the data obtained from the static positioning experiment in [17], the positioning error could be achieved 7.259 m (2*σ*) within the GDOP less than 1.5. From the experimental results of the static positioning experiment, it can be seen that the verification of the AIS Autonomous Positioning System (AAPS) [3] was performed to obtain high-accuracy positioning data.

## 5. Conclusions

In this paper, the effects of the seawater temperature, sea salinity, propagation distance, wind speed, and wave height on the ASFs, and the range positioning error corrections of the AIS reflected, diffracted signal and received signal are investigated. The ASFs of the AIS reflected signal is most affected by the propagation distance. The sea salinity and the wind speed have no impact on the ASFs of the AIS reflected signal for the AIS reflected signal. The seawater temperature has a slight effect on the ASFs of the AIS reflected signal. However, if the AIS signal is to be used for highly-accurate range positioning, the influence of the seawater temperature on the ASFs should be considered in order to obtain smaller positioning errors. The clearance parameter of the AIS diffracted signal varies with the wave height. At the same sea-wave position, the higher the wave height, the smaller the clearance parameter. The ASFs of the AIS diffracted signal show a trajectory of decrease first, increase then, decrease again and increase then with the increase of the clearance parameters. However, as for the wave height of 2 m, there will be a decreasing and then increasing oscillation after the same trend as the other wave heights. At the same clearance parameter, the ASFs of the AIS diffracted signal decreases with the increase of the wave height. For different wave heights, the ASFs of the AIS diffracted signal feature same changing trend. They have basically same maximum value and minimum value. The maximum ASF of the AIS diffracted signal over a rough sea surface appear at the maximum value point and minimum value point of the clearance parameter. The ASFs of the AIS received signal and the range positioning error correction increase with increasing the propagation distance. For the same clearance parameter, the ASFs and the range positioning error corrections of the AIS received signal increase with increases in the seawater temperature, the sea salinity, the wind speed, and the wave height. For the same seawater temperature, the ASFs and the range positioning error corrections of the AIS received signal present twice-upward trend with increasing the clearance parameter. Similar trends also present at the same sea salinity, wind speed and wave height points. The verification results show that after “coarse tuning” corrected by the additional secondary phase correction system for AIS signals, a positioning accuracy of 7.259 m (2*σ*) of the AAPS “fine-tuned” by the ASFs of the AIS received signal could be achieved with a GDOP of less than 1.5, which is superior to the positioning accuracy of 9.098 m (2*σ*) that uses solely the additional secondary phase correction system when GDOP is less than 1.5. Since research on the ASFs of the AIS received signal transmitted over a rough sea surface has not yet been conducted, the results of this paper are significant and serve as a reference for future research on high-precision position measurements.

## Figures and Tables

**Table 1:** The corresponding data change table of Figure 9.

Wave Height (m)	2	3	4	5	6	7
**Clearance Parameter (m)**	18–46.38	17–45.38	16–44.38	15–43.38	14–42.38	13–41.38
**ASF**	19.47–19.78	19.47–19.79	19.47–19.8	19.47–19.79	19.47–19.79	19.47–19.76
